# Sporadic adamantinomatous craniopharyngioma with double-hit somatic APC mutations

**DOI:** 10.1093/noajnl/vdab124

**Published:** 2021-08-30

**Authors:** Christopher S Hong, Antonio Omuro, Yi An, Silvio E Inzucchi, Anita A Kohli, Declan McGuone, Eugenia M Vining, Sacit Bulent Omay, E Zeynep Erson-Omay

**Affiliations:** 1 Department of Neurosurgery, Yale School of Medicine, New Haven, Connecticut, USA; 2 Division of Neuro-Oncology, Department of Neurology, Yale School of Medicine, New Haven, Connecticut, USA; 3 Department of Therapeutic Radiology, Yale School of Medicine, New Haven, Connecticut, USA; 4 Section of Endocrinology, Department of Medicine, Yale School of Medicine, New Haven, Connecticut, USA; 5 Department of Ophthalmology and Visual Science, Yale School of Medicine, New Haven, Connecticut, USA; 6 Department of Pathology, Yale School of Medicine, New Haven, Connecticut, USA; 7 Division of Otolaryngology, Department of Surgery, Yale School of Medicine, New Haven, Connecticut, USA

**Keywords:** adamantinomatous, APC, craniopharyngioma, CTNNB1

Craniopharyngiomas are benign neuroepithelial tumors of the suprasellar region. Histologically, there are adamantinomatous and papillary histological variants. Mutations in *CTNNB1*^1^ and *BRAF*^2^ characterize adamantinomatous and papillary subtypes, respectively. However, approximately up to 30% of adamantinomatous and 10% of papillary craniopharyngiomas do not exhibit these mutations, suggesting other mechanisms of tumorigenesis.^3,4^ There is a lack of understanding in the pathogenesis of craniopharyngiomas that are wildtype for both *CTNNB1* and *BRAF*, the majority of which are of adamantinomatous histopathology.^3^ In addition, while *BRAF*-mutated tumors may be amenable to targeted therapies with currently approved small molecule inhibitors,^5^ there are no readily available therapies for *CTNNB1*-mutated craniopharyngiomas, underlying a need for a better understanding of the biology of these tumors.

Previously, a few case reports have described adamantinomatous craniopharyngioma formation in patients with familial adenomatous polyposis (FAP), characterized by germline mutations in *APC*.^6–8^ Loss of this tumor suppressor gene leads to constitutive activation of the Wnt signaling pathway, similar to the observed downstream effects of activating *CTNNB1* mutations in adamantinomatous craniopharyngioma. In this report, we describe the first case, to our knowledge, of adamantinomatous craniopharyngioma arising from somatic loss of *APC* in the absence of germline mutations or a *CTNNB1* mutation.

## Methods

This study was conducted under an institutional review board-approved protocol at Yale University. The patient’s blood and tumor tissue were collected after obtaining written informed consent. Histopathology, including immunohistochemical studies, was evaluated by a board-certified neuropathologist.

Whole-exome sequencing (WES) and analysis was performed in accordance with our previously described methods at the Yale Cancer for Genome Analysis (YCGA).^9^ Briefly, genomic DNA from the tumor and blood were isolated and exome captured with IDT xGen Exome Research Panel v1 (Integrated DNA Technologies, Coralville, IA, USA) and then sequenced on the Illumina NovaSeq6000 WES platform with 2 × 100 base pair reads. Downstream analysis of raw reads, including alignment, duplicate marking, realignment, and base quality recalibration was performed according to “GATK Best Practice” recommendations (GATK v4.1.9). Somatic single-nucleotide variants (SNV), insertions/deletions (INDEL), and copy number variations (CNV) were identified as previously described.^9^ Mean coverage of 129.1× was achieved for blood and 224.9× for tumor tissue.

## Results

### Case Description

**Figure 1.**
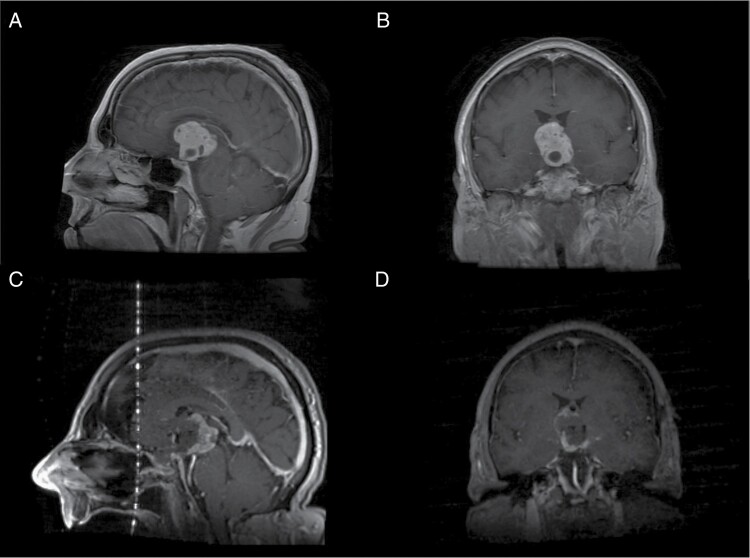
Preoperative and intraoperative MRI of the suprasellar lesion. Preoperative T1-weighted post-contrast MRI in the (A) sagittal and (B) coronal views demonstrate a contrast-enhancing lesion, measuring 3.8 × 3.0 × 3.5 cm. After subtotal resection via an endoscopic endonasal approach, intraoperative T1-weighted post-contrast MRI in the (C) sagittal and (D) coronal views demonstrate expected residual enhancing tumor left along the posterior aspect of the capsule, adherent to the hypothalamus.

A 74-year-old male with no relevant past medical history was initially evaluated by a neurologist for several months of short-term memory loss, difficulty taking medications, and personality changes, characterized by increased impulsivity and verbal aggression. As a part of his work-up, magnetic resonance imaging (MRI) of the brain was obtained, which demonstrated a contrast-enhancing suprasellar and third ventricular mass, measuring 3.8 × 3.0 × 3.5 cm (Figure 1A and B). On further review of systems, the patient admitted to increased thirst, fluid intake, and urination over the past year, including awakening multiple times per night to void. He also reported a 30-pound weight gain in the past year. On examination, he appeared hypogonadal but otherwise well with normal vital signs. Relevant laboratory values were notable for mild hyperprolactinemia (felt to be due to stalk effect and not primary production from the tumor), severe hypogonadotropic hypogonadism, mild central hypothyroidism, and low insulin-like growth factor-1 indicative of growth hormone deficiency. There was no evidence of adrenal insufficiency, and urinary concentrating ability appeared preserved, arguing against central diabetes insipidus, despite his history.

On neuro-ophthalmic examination, visual acuity was 20/30 in the right eye and 20/25 in the left eye. Color vision was full, and there was no afferent pupillary defect. There was mild optic neuropathy of the left eye, characterized by trace temporal pallor. Humphrey visual field showed inferotemporal losses in the left eye only, and optical coherence tomography of the retinal nerve fiber layer revealed left greater than right temporal nerve fiber layer thinning.

**Figure 2.**
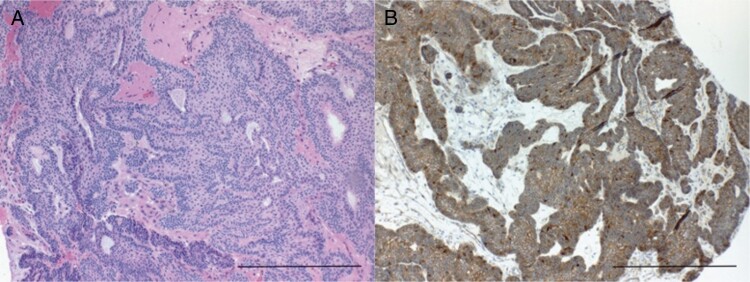
Histopathology of the tumor tissue. (A) Hematoxylin and eosin stain shows an adamantinomatous craniopharyngioma with cystic spaces, basal palisading, stellate reticulum, and wet keratin (×200 magnification, scale bar = 20 μM). (B) Immunohistochemistry for beta-catenin demonstrates focal areas of nuclear expression (×200 magnification, scale bar = 20 µM).

Given the size of the lesion and patient’s symptomatology, surgical resection was advised. Subsequently, the patient underwent an endoscopic endonasal approach for resection of the lesion, utilizing the chiasm-pituitary corridor. Residual tumor, adherent within the posterior aspect of the capsule was intentionally left to avoid injury to the hypothalamus. An intraoperative MRI was obtained, revealing significant decompression of the optic apparatus with expected residual disease along the posterior confines of the tumor field (Figure 1C and D). Histopathology of the tumor confirmed a World Health Organization (WHO) grade I craniopharyngioma of adamantinomatous subtype (Figure 2A) with focal nuclear expression of beta-catenin (Figure 2B).

Postoperatively, the patient did develop diabetes insipidus as well as secondary hypoadrenalism and has been treated with daily oral desmopressin and prednisone. Levothyroxine, which had been started preoperatively, is being continued. Hypogonadism persists and androgen replacement therapy is being considered as is, potentially, human growth hormone replacement therapy. His cognitive symptoms have improved. He is planned to undergo radiation therapy to his known residual tumor.

### Genomic Analysis

WES was performed on the resected surgical specimen and matched blood in accordance with an institutional review board-approved protocol and utilizing previously described methods.^10^ The analysis of somatic SNV/INDEL data revealed 2 distinct somatic stop codon mutations in *APC*, (rs786201856: c.C793T:p.R265X and rs587779780: c.1159T:p.R387X). Interestingly, both mutations displayed similar high variant allele frequency (VAF) of 39.9% and 45.4%, respectively, indicating that both alterations were indeed clonal, co-occurring in the majority of the cell population. Both variants have been reported in patients with sporadic and syndromic adenocarcinomas within the COSMIC database^11^ and are considered pathogenic by ClinVar.^12^ Detailed data are provided in Table 1. There were no somatic CNV or loss of heterozygosity events. Notably, no somatic mutations were detected in *CTNNB1* or *BRAF*. Likewise, no germline mutations were found to suggest an underlying cancer predisposition syndrome, consistent with the patient’s unremarkable personal and familial past medical histories.

**Table 1.** Genetic Findings of Index Patient

Gene	Chromosome	Position	Ref	Alt	HGVS (RefGene)	dbSNP
APC	5	112151204	C	T	NM_001127511:exon7:c.C793T:p.R265X	rs786201856
APC	5	112154942	C	T	NM_001127511:exon8:c.C1159T:p.R387X	rs587779780

## Discussion

The majority of adamantinomatous craniopharyngiomas demonstrate somatic mutations in *CTNNB1*,^1^ which encodes beta-catenin, a protein that acts to transduce intracellular signals in the Wnt signaling pathway. *CTNNB1* is considered a proto-oncogene, as oncogenic mutations prevent the degradation of beta-catenin, leading to constitutive activation of the Wnt pathway and downstream effects of increased cellular proliferation and migration.^13^ In contrast, *APC* is classified as a tumor suppressor gene, whose protein product APC functions to bind and subsequently target beta-catenin in the cytoplasm for degradation.^14^ As such, loss of APC function similarly leads to constitutive activation of beta-catenin-driven Wnt signaling.^13^

*APC* mutations are most commonly seen in colon cancers. A specific subset of patients harbor germline mutations, leading to FAP, an autosomal dominant condition characterized by innumerable adenomatous polyp formation in the large intestine with high potential for malignant transformation. While rare, craniopharyngiomas have been reported in 10 patients with a known diagnosis of FAP, comprised of 7 ectopic cases (located in the cerebellopontine angle) and 3 in the sellar region.^6–8,15^ However, further genetic studies in these reports have been limited. Dahl et al described a *CTNNB1* mutation in a sellar craniopharyngioma, in addition to the known germline *APC* mutation but did not find a second hit to *APC* to suggest a role for *APC* loss in tumor formation.^15^

In contrast, only a few reports of craniopharyngiomas, arising from complete loss of APC through a germline and a second-hit somatic mutation and in the absence of *CTNNB1* mutations, have been reported to date. Gorelyshev et al described 2 cases of sellar craniopharyngiomas in a pair of half-siblings without a known diagnosis of FAP.^7^ WES revealed a previously undescribed germline *APC* variant in both patients, as well as shared somatic *APC* mutations affecting a known hotspot region in their tumors. Biallelic loss of APC could only be confirmed in 1 patient utilizing linked heterozygous SNP markers to differentiate between alleles, but the same approach could not be used for the second patient who was homozygous for the same markers. Notably, despite germline findings, a clinical diagnosis of FAP could not be made, as 1 sibling had a normal colonoscopy, while the other refused the procedure. While this study implicated loss of APC in craniopharyngioma formation, direct causality remained unclear due to the uncertain pathogenicity of the germline *APC* variant and inability to demonstrate biallelic loss of APC in both patients. Further evidence came from Passos et al who described a patient with FAP from a known pathogenic *APC* germline variant who underwent resection of an ectopic craniopharyngioma in the cerebellopontine angle.^8^ In addition to the germline mutation, the tumor carried a second somatic variant affecting the other allele, leading to a premature stop codon, and subsequently biallelic loss of APC.

Here, we describe the first case to our knowledge of craniopharyngioma formation, driven by loss of APC function secondary to 2 independent somatic mutations rather than germline pathogenic variants. Although our WES methodology was limited in its ability to determine whether the 2 stop codon *APC* mutations occurred on separate alleles, immunohistochemical staining for beta-catenin demonstrated pathologic nuclear localization, characteristic of beta-catenin overexpression and seen in *CTNNB1*-mutated craniopharyngiomas. Furthermore, we can conclude from the high VAF of the 2 *APC* mutations that they were both present in the majority of the cell population, further supporting the notion of biallelic loss. Considering the genetic profile of our patient’s tumor, the results of immunohistochemical staining were also consistent with loss of APC function, driving pathologic nuclear beta-catenin activity.

Therapeutic targeting of aberrant beta-catenin activity remains an intense focus in cancer research. However, as Wnt signaling is a highly conserved pathway essential to normal cellular physiology, the development of drugs targeting cancer cells with acceptable safety profiles has remained a pharmacologic challenge. Several drugs that inhibit upstream effectors in the Wnt signaling pathway have undergone early clinical trial testing, albeit with concerns for off-target effects. Vantictumab, a monoclonal antibody against Fzd receptors that prevents binding with all Wnt ligands to suppress Wnt signaling, demonstrated moderate efficacy in a phase 1 clinical trial with paclitaxel in Wnt-upregulated metastatic breast^16^ and pancreatic^17^ cancers, but concerns around bone-related safety have been raised. In addition, inhibitors against porcupine, an enzyme involved in the processing of Wnt signaling proteins, have been developed to target Wnt-driven cancers, most notably with WNT974, which has shown safety but limited efficacy in advanced solid tumors.^18^ However, issues remain surrounding the efficacy of targeting upstream targets, in regard to resistance in tumors with more downstream mutations, such as *CTNNB1*- or *APC*-mutated cancers.^19^ Currently, direct inhibition of beta-catenin or APC remains a pharmacologic challenge limited to preclinical testing^19,20^ and significant concerns for off-target effects remain, such as toxicity to normal intestinal tissues.^21^ As such, further work is needed to develop safe but effective inhibitors of aberrant Wnt signaling in cancer.

This study provides evidence that somatic loss of APC can lead to sporadic craniopharyngioma formation and may comprise a subset of adamantinomatous craniopharyngiomas that are otherwise wildtype for *CTNNB1*. Additionally, while it has been suggested that APC-driven craniopharyngiomas may have a predilection for ectopic formation,^15^ our case demonstrates that typical, sellar craniopharyngiomas may also arise from loss of APC. Further genomic analyses are needed to determine whether genetic differences exist between ectopic and sellar craniopharyngiomas.

## Conclusion

Taken together, this case demonstrates that adamantinomatous craniopharyngiomas, typically driven by proto-oncogenic *CTNNB1* mutations, may also arise independently from loss of the tumor suppressor gene *APC*, leading to similar downstream constitutive activation of the Wnt signaling pathway. In adamantinomatous craniopharyngiomas that are wildtype for *CTNNB1* in targeted screens, *APC* sequencing may be considered. Additional genetic studies are needed to determine whether significant biological differences exist in APC- vs CTNNB1-driven craniopharyngiomas that may guide further clinical decision making and development of target therapies.

## References

[1] Buslei R, Nolde M, Hofmann B, et al. Common mutations of beta-catenin in adamantinomatous craniopharyngiomas but not in other tumours originating from the sellar region. Acta Neuropathol. 2005;109(6):589–597. 15891929 10.1007/s00401-005-1004-x

[2] Brastianos PK, Taylor-Weiner A, Manley PE, et al. Exome sequencing identifies BRAF mutations in papillary craniopharyngiomas. Nat Genet. 2014;46(2):161–165. 24413733 10.1038/ng.2868 PMC3982316

[3] Omay SB, Chen YN, Almeida JP, et al. Do craniopharyngioma molecular signatures correlate with clinical characteristics? J Neurosurg. 2018;128(5):1473–1478. 28707994 10.3171/2017.1.JNS162232

[4] Prieto R, Pascual JM. Can tissue biomarkers reliably predict the biological behavior of craniopharyngiomas? A comprehensive overview. Pituitary. 2018;21(4):431–442. 29700680 10.1007/s11102-018-0890-6

[5] Juratli TA, Jones PS, Wang N, et al. Targeted treatment of papillary craniopharyngiomas harboring BRAF V600E mutations. Cancer. 2019;125(17):2910–2914. 31314136 10.1002/cncr.32197 PMC7032527

[6] Gabel BC, Cleary DR, Martin JR, Khan U, Snyder V, Sang UH. Unusual and rare locations for craniopharyngiomas: clinical significance and review of the literature. World Neurosurg. 2017;98:381–387. 27908738 10.1016/j.wneu.2016.10.134

[7] Gorelyshev A, Mazerkina N, Medvedeva O, et al. Second-hit APC mutation in a familial adamantinomatous craniopharyngioma. Neuro Oncol. 2020;22(6):889–891. 32170310 10.1093/neuonc/noaa060 PMC7283019

[8] Passos J, Quidet M, Brahimi A, et al. Familial adenomatous polyposis associated craniopharyngioma secondary to both germline and somatic mutations in the APC gene. Acta Neuropathol. 2020;140(6):967–969. 33025138 10.1007/s00401-020-02232-9

[9] Fomchenko EI, Erson-Omay EZ, Zhao A, et al DNMT3A co-mutation in an IDH1-mutant glioblastoma. Cold Spring Harb Mol Case Stud. 2019;5(4):a004119. 10.1101/mcs.a004119 PMC6672028 31371348

[10] Hong CS, Kuzmik GA, Kundishora AJ, et al. Hypermutated phenotype in gliosarcoma of the spinal cord. NPJ Precis Oncol. 2021;5(1):8. 33580181 10.1038/s41698-021-00143-w PMC7881101

[11] Tate JG, Bamford S, Jubb HC, et al. COSMIC: the catalogue of somatic mutations in cancer. Nucleic Acids Res. 2019;47(D1):D941–D947. 30371878 10.1093/nar/gky1015 PMC6323903

[12] Landrum MJ, Lee JM, Benson M, et al. ClinVar: improving access to variant interpretations and supporting evidence. Nucleic Acids Res. 2018;46(D1):D1062–D1067. 29165669 10.1093/nar/gkx1153 PMC5753237

[13] Bugter JM, Fenderico N, Maurice MM. Mutations and mechanisms of WNT pathway tumour suppressors in cancer. Nat Rev Cancer. 2021;21(1):5–21. 33097916 10.1038/s41568-020-00307-z

[14] Rubinfeld B, Albert I, Porfiri E, Fiol C, Munemitsu S, Polakis P. Binding of GSK3β to the APC-β-catenin complex and regulation of complex assembly. Science. 1996;272(5264):1023–1026. 8638126 10.1126/science.272.5264.1023

[15] Dahl NA, Pratt D, Camelo-Piragua S, et al. Pediatric craniopharyngioma in association with familial adenomatous polyposis. Fam Cancer. 2019;18(3):327–330. 30919136 10.1007/s10689-019-00126-8 PMC7504906

[16] Diamond JR, Becerra C, Richards D, et al. Phase Ib clinical trial of the anti-frizzled antibody vantictumab (OMP-18R5) plus paclitaxel in patients with locally advanced or metastatic HER2-negative breast cancer. Breast Cancer Res Treat. 2020;184(1):53–62. 32803633 10.1007/s10549-020-05817-w PMC7572714

[17] Davis SL, Cardin DB, Shahda S, et al. A phase 1b dose escalation study of Wnt pathway inhibitor vantictumab in combination with nab-paclitaxel and gemcitabine in patients with previously untreated metastatic pancreatic cancer. Invest New Drugs. 2020;38(3):821–830. 31338636 10.1007/s10637-019-00824-1 PMC7211194

[18] Rodon J, Argilés G, Connolly RM, et al. Phase 1 study of single-agent WNT974, a first-in-class Porcupine inhibitor, in patients with advanced solid tumours. Br J Cancer. 2021;125(1):28–37. 33941878 10.1038/s41416-021-01389-8 PMC8257624

[19] Wang Z, Li Z, Ji H. Direct targeting of β-catenin in the Wnt signaling pathway: current progress and perspectives. Med Res Rev. 2021;41(4):2109–2129. 33475177 10.1002/med.21787 PMC8217106

[20] Zhang L, Theodoropoulos PC, Eskiocak U, et al Selective targeting of mutant adenomatous polyposis coli (APC) in colorectal cancer. Sci Transl Med. 2016;8(361):361ra140. 10.1126/scitranslmed.aaf8127 PMC7262871 27798265

[21] Kahn M. Can we safely target the WNT pathway? Nat Rev Drug Discov. 2014;13(7):513–532. 24981364 10.1038/nrd4233 PMC4426976

